# Draft Genome Sequence of the Broad-Spectrum Xenobiotic Degrader *Achromobacter xylosoxidans* ADAF13

**DOI:** 10.1128/genomeA.00203-16

**Published:** 2016-04-14

**Authors:** Rupa Iyer, Ashish Damania

**Affiliations:** a Center for Life Sciences Technology, College of Technology, University of Houston, Houston, Texas, USA; b Department of Pediatrics, Section of Pediatric Tropical Medicine, Baylor College of Medicine, Houston, Texas, USA

## Abstract

*Achromobacter xylosoxidans* ADAF13, isolated from farmland soil, possesses a large number of putative degradation genes and pathways that break down a wide variety of aromatic hydrocarbons, pesticides, endocrine disruptors, and other high-impact xenobiotics. These properties make this strain an excellent candidate for further development as a broad-spectrum bioremediation agent.

## GENOME ANNOUNCEMENT

*Achromobacter xylosoxidans* ADAF13 is a Gram-negative, oxidase- and catalase-positive bacterium from the genus *Achromobacter*. *A. xylosoxidans* is well known for its opportunistic pathogenicity and infection of pulmonary tissue in immunocompromised individuals. These infections are particularly common in cystic fibrosis patients (1). However, *A. xylosoxidans* is a metabolically versatile microorganism and has significant potential value in environmental bioremediation applications (1). We have isolated and identified a new strain of *A. xylosoxidans* from farmland soil taken from Cypress, TX, as part of an undergraduate environmental sampling research module that collects samples from across the state of Texas and screens them for bacteria with the capacity to degrade organophosphate insecticides (2). In comparison to other *Achromobacter* genome projects, genomic analysis showed that ADAF13 is most closely related to *Achromobacter arsenitoxydans* SY8, and then *Achromobacter piechaudii* HLE, *Achromobacter* sp. strain DH1f, and *A. xylosoxidans* C54, in order of decreasing similarity. While lacking the extensive arsenite gene islands that define *A. arsenitoxydans* SY8 (3), we report here the genome sequence of a broad-spectrum xenobiotic degrader of polycyclic aromatic hydrocarbons and organophosphate insecticides with the added capacity to putatively target both bisphenol A and trinitrotoluene. The genome sequencing of ADAF13 was performed through Illumina MiSeq paired-end sequencing (total reads, 3,363,665; 35 to 250 bp in each read), with a final sequencing coverage of 194.86×. Sequence reads were checked for quality using FastQC (http://www.bioinformatics.babraham.ac.uk/projects/fastqc/) and filtered using BBTools (https://sourceforge.net/projects/bbmap/), with a minimum Phred score of 20. Paired-end reads were assembled into 120 contigs with the SPAdes 3.6.2 program (4). Preliminary reference-based annotation using PATRIC (5) Web resources was carried out to identify conserved pathways. Final *de novo* annotation was performed with Prokka (6) and the NCBI Prokaryotic Genome Annotation Pipeline (http://www.ncbi.nlm.nih.gov/genomes/static/Pipeline.html). The metabolic pathways of aromatic and heterocyclic compounds were examined using the KEGG databases (7). ADAF13 has a G+C content of 65.84% and contains 5,184 putative coding sequences (CDSs; 984 bp average length), of which 4,021 CDSs are functional. The project accession also contains sequences for 6 rRNA, 54 tRNA, and 4 noncoding RNA (ncRNA) loci.

### Nucleotide sequence accession numbers.

The *A. xylosoxidans* ADAF13 whole-genome shotgun (WGS) project has the project accession no. LSMI00000000. This version of the project (01) has the accession no. LSMI01000000 and consists of sequences LSMI01000001 to LSMI01000120.
